# Identification of SNPs Related to *Salmonella* Resistance in Chickens Using RNA-Seq and Integrated Bioinformatics Approach

**DOI:** 10.3390/genes14061283

**Published:** 2023-06-17

**Authors:** Mashooq Ahmad Dar, Basharat Bhat, Junaid Nazir, Afnan Saleem, Tasaduq Manzoor, Mahak Khan, Zulfqarul Haq, Sahar Saleem Bhat, Syed Mudasir Ahmad

**Affiliations:** 1 Division of Animal Biotechnology, Faculty of Veterinary Sciences and Animal Husbandry, Shuhama, SKUAST-Kashmir, Srinagar 190006, India; darmashooq1@gmail.com (M.A.D.); junaidnaxir@gmail.com (J.N.); afnankhan1082@gmail.com (A.S.); tasaduqmanzoor88@gmail.com (T.M.); khanmahak955@gmail.com (M.K.); 2 Laboratory of Preclinical Testing of Higher Standard, Nencki Institute of Experimental Biology of Polish Academy of Sciences 3, 02-093 Warsaw, Poland; 3 Department of Clinical Biochemistry, Lovely Professional University, Phagwara 144402, India; 4 Indian Council of Medical Research Project, Division of Livestock Production and Management, F.V.Sc & AH, Shuhama, Sher-e-Kashmir University of Agricultural Sciences and Technology of Kashmir, Srinagar 190006, India; zulfy11@gmail.com; 5 Genomics Lab, ABT, FVSC & AH, SKUAST-Kashmir, Srinagar 190006, India

**Keywords:** *Kashmir favorella*, broiler, SNP identification, RNA sequencing

## Abstract

Potential single nucleotide polymorphisms (SNPs) were detected between two chicken breeds (*Kashmir favorella* and broiler) using deep RNA sequencing. This was carried out to comprehend the coding area alterations, which cause variances in the immunological response to Salmonella infection. In the present study, we identified high impact SNPs from both chicken breeds in order to delineate different pathways that mediate disease resistant/susceptibility traits. Samples (liver and spleen) were collected from *Salmonella* resistant (*K. favorella*) and susceptible (broiler) chicken breeds. *Salmonella* resistance and susceptibility were checked by different pathological parameters post infection. To explore possible polymorphisms in genes linked with disease resistance, SNP identification analysis was performed utilizing RNA seq data from nine *K. favorella* and ten broiler chickens. A total of 1778 (1070 SNPs and 708 INDELs) and 1459 (859 SNPs and 600 INDELs) were found to be specific to *K. favorella* and broiler, respectively. Based on our results, we conclude that in broiler chickens the enriched pathways mostly included metabolic pathways like fatty acid metabolism, carbon metabolism and amino acid metabolism (Arginine and proline metabolism), while as in *K. favorella* genes with high impact SNPs were enriched in most of the immune-related pathways like MAPK signaling pathway, Wnt signaling pathway, NOD-like receptor signaling pathway, etc., which could be a possible resistance mechanism against salmonella infection. In *K. favorella*, protein–protein interaction analysis also shows some important hub nodes, which are important in providing defense against different infectious diseases. Phylogenomic analysis revealed that indigenous poultry breeds (resistant) are clearly separated from commercial breeds (susceptible). These findings will offer fresh perspectives on the genetic diversity in chicken breeds and will aid in the genomic selection of poultry birds.

## 1. Introduction

*Salmonella enterica* serovar *typhimurium* is a Gram-negative, facultative anaerobe, non-spore producing, motile bacillus in the Enterobacteriaceae family. It colonizes the digestive tracts of many vertebrates and cause severe intestinal pathology in young chicken [1,2]. Salmonellosis poses a serious socioeconomic hazard and is associated with significant human and animal mortality and morbidity [3]. *Salmonella* is one of the most prevalent bacteria, responsible for sporadic cases or outbreaks of gastroenteritis [4]. The annual economic cost of foodborne illness has been estimated to be as high as USD90 billion [5]. Globally, *Salmonella* is the most common cause of foodborne illnesses, with poultry and poultry-related products considered the primary causes of such outbreaks [6]. *Salmonella* infection is a major threat in the poultry industry. Poultry is a major global reservoir of non-typhoidal Salmonellae that cause foodborne diseases. Systemic salmonellosis causes significant losses in the poultry sector in terms of mortality and decreased poultry production [7]. Vaccination, sanitation, and the use of antibiotics are the most common methods used to combat *Salmonella* infections. As none of the current vaccination programs have been successful in controlling *Salmonella* infections [8], antibiotics are being preferred by the poultry industry. Owing to widespread antibiotic use, growth of antibiotic-resistant microorganisms and the buildup of antibiotic residues in food intended for human consumption are the two main problems resulting from the emergence of antibiotic-resistant bacteria and the accumulation of antibiotic residues in food. These are the two key difficulties associated with widespread antibiotic use in poultry for human consumption [9,10]. The human immune system has the power to successfully fend off microbial invasion and eradicate microbes. Following bacterial identification, host macrophages induce bactericidal action, which stimulates the maturation and migration of dendritic cells as well as the production of inflammatory chemokines, cytokines, interleukins, and other substances [11]. On the other hand, *Salmonella* has certain defense mechanisms that help it to combat these host barriers and inhibit host-immune responses via their its virulence genes [11]. After the Salmonella reaches the intestinal macrophages, it senses the phagosomal environment and triggers various virulence mechanisms that help it to survive in the macrophages [12]. *Salmonella* uses the Salmonella pathogenicity islands during host invasion. Within the Salmonella pathogenicity islands 1 and 2, *Salmonella* encodes two distinct virulence related T3SSs that act at different times during infection [13]. Once in contact with the host cell, the SPI1-encoded T3SS is activated and translocates bacterial proteins across the plasma membrane, while the SPI2-encoded T3SS that is expressed within phagosomes is involved in the translocation of the effectors across the vacuolar membrane. The SPI1 system plays a key role in invading non-phagocytic cells, induction and activation intestinal inflammatory responses, diarrhea and intestinal colonization. In contrast, the SPI2-encoded T3SS is required for the survival of bacteria in macrophages and the onset of systemic disease [12]. After the *Salmonella* has entered the cell, it resides within a vacuolar compartment known as a spacious phagosome (SP) [14]. This spacious phagosome shrinks within minutes to hours and forms an adherent membrane that wraps one or more bacteria and is referred as the *Salmonella*-containing vacuole (SCV). Intracellular persistence of SCV can range from hours to days, which makes it a unique phagosome in terms of normal maturation and recycling of phagolysosomes. Despite controversies, some studies report that *Salmonella* can live in macrophages that have lysosomal compartments fused with the SCV [15,16].

In the context of the above statements, genetic resistance is a long-term approach for a disease-control strategy [17]. Selecting more resistant hens may be an alternative option to reduce illness occurrence. Genetic disease resistance is often more relevant in underdeveloped nations since indigenous breeds are more resistant to local diseases [18]. *K. favorella* is a well-known indigenous chicken breed from the north Indian state Jammu and Kashmir. It is regarded as the most significant source of animal protein and is raised largely for meat and egg production [19]. The local climate conditions, feed, and stress management are well matched to this native breed, which has a good disease resistance [20].

Recent developments in molecular science have opened up new possibilities for improving quantitative traits genetically, especially those that are resistant to disease. The use of gene introgression or marker-assisted selection would be made easier with the discovery of direct or indirect molecular markers. Molecular markers are essential tools for marker-assisted breeding. The simple sequence repeats (SSRs) (SNP) markers are two attractive and widely used markers because of many merits including locus-specificity, reproducibility, co-dominance, and random genome-wide distribution in many organisms [21,22]. These features, however, are not only regulated at the DNA level, but also at the mRNA level before and after transcription, and this level of regulation is more extensive, systematic, and accurate. SNP detection by RNA-seq is of great interest to researchers as whole genome sequencing is expensive as well as exome sequencing tools are uncommon. The detection of SNPs in coding regions is used to understand variants affecting protein functions and analyze allele-specific expressions. Gene expression can be highly variable and which makes SNP detection and genotype calling by RNA-seq a challenging endeavor [23]. The study aims to identify SNPs, which are potentially involved in disease resistance against *Salmonella* infection in poultry and thus may lay a foundation step for future in-depth studies of disease resistance mechanisms.

## 2. Materials and Methods

### 2.1. Experimental Birds and Sample Collection

Experimentation and animal tissue collection was carried out with the proper consent of the Institutional Animal Ethics Committee on Ethical Standards in Animal Experiments (AU/FVSc/PS-57/16021). During all of the experimental studies, the Institutional Animal Ethics Committee’s rules were rigorously followed. The experimental chicks from two breeds, i.e., *K. faverolla* and broiler (Cobb), were procured from the Division of Livestock Production and Management, Sher-e-Kashmir University of Agricultural Sciences and Technology of Kashmir (SKUAST-K)-India. From day one of hatching, the experimental birds were kept in the animal house facilities center at SKUAST-K, under standard sanitary, temperature, and pressure conditions. The birds were monitored on a daily basis and provided unlimited access to food and water.

To ensure that all the birds under experimental study from both the breeds were free from *Salmonella* infection, fecal swabs from all the birds were taken and inoculated in tetrathionate broth and further streaked on BGA and MacConkey plates. Only *Salmonella* negative birds were taken for the study. After 12 h post infection, fecal swabs were taken from all the birds and incubated overnight in tetrathionate broth at 42 °C. The overnight culture was streaked on BGA and MacConkey plates. The plates were kept overnight at 37 °C. Following overnight incubation, the colonies on the plates were then examined for *Salmonella* species.

After giving an acclimatization time of 3 days to the chicks from both the breeds, they were orally infected with *S. typhimurium* (2 × 10^8^ CFU/mL) at 4 days of age and were initially assessed for disease resistance up to 10 days post infection. The two chicken breeds were classified as *Salmonella*-resistant and -susceptible breeds by taking into consideration the clinical symptoms and bacterial loads. The clinical scores were recorded twice daily following points based a scoring system. Chicks with severe clinical signs (progressive weakening, anorexia, diarrhea, and head lowering) as well as significant liver disease and greater bacterial loads in fecal swabs were identified as the challenged susceptible group. The challenged-resistant birds were recognized as chicks with little clinical and pathological symptoms and low bacterial burdens. The *K. favorella* was determined to be resistant based on the aforementioned clinical signs and bacterial levels, and broilers were determined to be susceptible to *Salmonella* infection. Samples (liver and spleen) were collected from *Salmonella*-resistant (*K. favorella)* and susceptible (broiler) chicken breeds [1] (Table S1).

Carcass of sacrificed birds were subjected to a systemic necropsy technique for the examination and documentation of the *Salmonella* specific lesions. Lesions primarily included bronze colored discoloration of liver, typhilitis and splenomegaly. For comparative histopathological analysis, representative liver and spleen samples from commercial broilers and *K. favorella* were collected in 10% buffered formalin and processed using the standard paraffin embedding technique using alcohol and acetone as dehydrating agents, benzene as a clearing agent, and paraffin wax with a melting point of 60 °C. For routine investigation, sections of 5 m thickness were cut and stained with Harris Hematoxylin and Eosin.

In this study, 10 samples from broiler chicken (5 liver and 5 spleen) and 9 samples from favorella chicken (5 liver and 4 spleen) were utilized to identify SNPs that could potentially mediate the *Salmonella* disease resistance in chicken. To reduce the effect of confounding factors, and increase sensitivity and specificity of SNPs, both groups have control and infected samples. In the broiler liver group, 3 are infected and 2 are control samples. Similarly, in the broiler spleen group, 3 are infected and 2 are control samples. In the *K. favorella* liver group, 2 are infected and 2 are control samples. In the *K. favorella* spleen group, 3 are infected and 2 are control samples. The reason for including infected samples with control for SNP identification was to reduce any biological, technical or genomic factors that could possibly affect the key SNP identification with the phenotype of interest, i.e., *Salmonella* resistance.

### 2.2. Total RNA Isolation, cDNA Library Construction, and Sequencing

The sequencing data were downloaded from our previously published dataset NCBI (Accession ID: GSE168060). All sample processing and sequencing steps are described previously [24]. Briefly, total RNA was extracted using Trizol method (Ambion, Naugatuck, CT, USA) following the manufacturers guidelines. The RNA quality and integrity were examined using a spectrophotometer (ThermoFisher, Waltham, MA, USA) and a bioanalyzer (Agilent, Santa Clara, CA, USA). Libraries were prepared using RNA samples with RIN values ≥ 8. The Illumina TruSeq stranded mRNA sample preparation kit was used to construct cDNA libraries, and the manufacturer’s protocol was followed. The 4 μg/sample total RNA was utilized to prepare libraries. Poly-T attached magnetic beads were used to purify poly-A containing mRNA molecules. Following purification, divalent cations were used in a high-temperature process to break the RNA down into smaller bits. Using the enzyme reverse transcriptase and random primers, the RNA fragments were utilized to create first strand cDNA (Illumina, San Diego, CA, USA). After DNA fragments were adenylated (at their 3 ends), the hybridization process was started by ligating the Illumina paired-end adaptor and index. Using an Illumina PCR primer cocktail, the cDNA fragments (150 bp) were produced and used to create the sequencing paired end cDNA library. Libraries were pooled in equimolar levels using a High Throughput Model flow cell on an Illumina HiSeq 2500 platform and paired end sequenced by SciGenom, Cochin, Kerala-India.

### 2.3. Quality Control, Aligning and Mapping Reads to the Genome

The FASTQC program v0.11.1 was used to examine read quality control [25]. Following preprocessing, low-quality sequence filtering and adaptor trimming with Cutadapt v3.40 [26], high-quality sequencing reads that exceeded thresholds (Phred Score > 30) were combined for SNP identification analysis. For each sample, more than 40 million clean, high-quality readings were gathered. HISAT2 was used to map the cleaned reads to the reference genome assembly ARS-UCD1.2.99 [27]. Before variant identification, the data pretreatment stages suggested in the Genome Analysis Toolkit (GATK) best practices workflow were carried out [28]. MarkDuplicates from Picard tools were used to identify PCR duplicates [29]. Additionally, we used GATK to recalibrate the base quality scores, examine intron–exon junctions, and perform local realignment around InDels. SNP and INDELs discovery across 10 broiler and 10 *K. favorella* transcriptome samples independently was carried out using two distinct variant callers: (i) mpileup from SAMtools v1.4 [30] in multi-sample calling mode with default parameters; (ii) GATK utilizing the HaplotypeCaller tool in multi-sample calling mode (modality “GATK”). The final set for analysis contains SNPs and InDels, which are common in both datasets. Chicken genetic variants from dbSNP 2.0 build 153 dated: 8 August 2019 were incorporated in SNP calling to populate the RS_ID column of the known SNPs. Filtering [base quality score (Q Score) > 30, mapping quality > 30, and minimum depth > 10] of generated variants and annotation were performed using VCFtools version 0.1.8 and SnpEff program v4.1. Biological significance was further evaluated for the genes that had high-impact variations. The KEGG pathway enrichment analysis was performed using KOBAS server version 3 [31,32].

## 3. Results

### 3.1. Quality Control, Mapping, and Post Treatment

**Figure 1:**
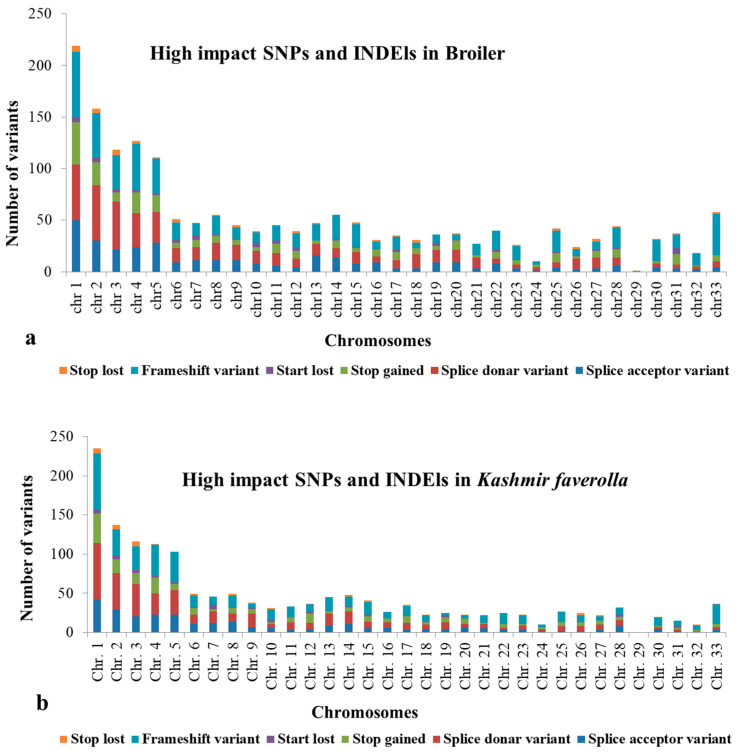
Chromosomal distribution of high impact SNPs and Indels in (**a**) Broiler chicken and (**b**) *K. favorella*.

**Figure 2:**
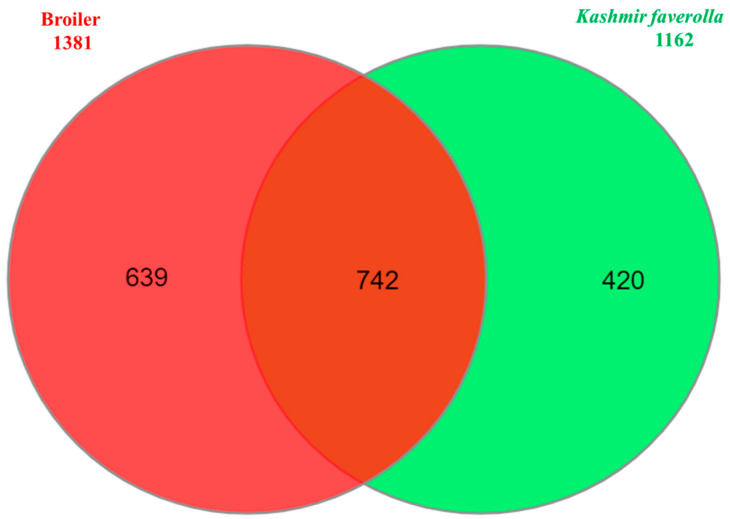
Venn diagram showing common genes with SNPs between *K. favorella* and broiler chicken breeds.

A total of 46.95 million reads (range 30.51–68.56 million reads/library) and 41.74 million reads (range 26.92–63.61 million reads/library) were generated by the liver and spleen transcriptome libraries, respectively. Overall, 43.76 million reads (92.82%) of the 46.95 million hepatic transcriptome reads passed quality control and were mapped to the *Gallus gallus* genome GRCg6a. The remaining reads were deleted, and 41.46 million uniquely mapped reads in total were processed further. In the spleen, 38.65 million reads (92.24%) of the total 41.74 million reads passed the quality check and were aligned to the *G. gallus* CRCg6a genome. Additionally, 35.45 million uniquely mapped reads were processed further, while the remaining reads were discarded. *K. favorella* and broiler yielded a total of 1,141,122 and 1,151,874 variations, respectively. The chromosomal distribution of SNPs and INDELs is shown in Table S2 and the variant types are shown in Tables S3 and S4. Kashmir favorella had a total of 26.036% missense, 0.138% nonsense, and 73.785% silent alterations, while broiler chicken had a total of 26.845% missense, 0.167% nonsense, and 72.988% silent mutations. The transitions to transversions ratio (Ts/Tv) for *K. favorella* and broiler was determined to be 2.7 (6080998/2236247) and 2.7 (6993925/2563136), respectively, in line with earlier studies. SNP distribution on different chromosomes in both *K. favorella* and commercial broiler are shown (Figure 1). It was found that there were 758 common genes with SNPs (Figure 2). The common SNPs were filtered out, and the high-impact SNPs and INDELs specific to broiler 1778 (1070 SNPs and 708 INDELs) and *K. favorella* 1459 (859 SNPs and 600 INDELs) (Supplementary File S1 and S2) were further studied. List of genes with high impact SNPs involved in *Salmonella* disease resistance in chickens are shown in Table 1.

### 3.2. Analysis of Genes with SNPs and INDELs

**Figure 3:**
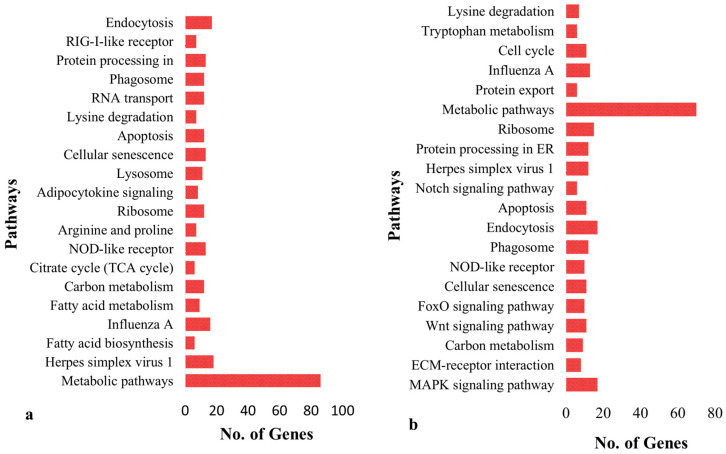
KEGG enrichment of genes with high impact variations. (**a**) Broiler. (**b**) *K. favorella*.

**Figure 4:**
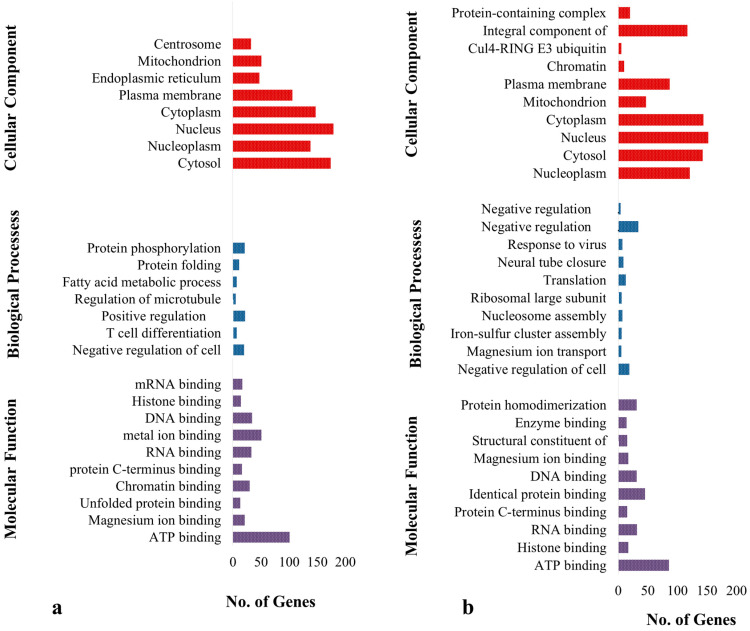
Gene ontology analysis of genes with high impact SNPs and INDELs. (**a**) Broiler. (**b**) *K. favorella.*

Functional annotation indicates that signaling pathways, such as those involved in metabolism, herpes simplex virus type 1 infection, Influenza A, fatty acid biosynthesis, fatty acid metabolism, carbon metabolism and citrate cycle in broiler chicken are enriched (*p* < 0.05) (Table S5, Figure 3a). The enhanced pathways in *K. favorella* chicken comprised the Wnt signaling route, the FoxO signaling pathway, the cellular senescence pathway, and the NOD-like receptor signaling pathway (Table S6, Figure 3b). In *K. favorella* and broiler chicken, gene ontology (GO) research shows genes (*p* < 0.05) with SNPs were primarily involved in the binding process (enzyme, ribonucleotide, and histone binding), as well as antigen processing (Figure 4a,b, Tables S7 and S8).

### 3.3. Protein-Protein Interaction

**Figure 5:**
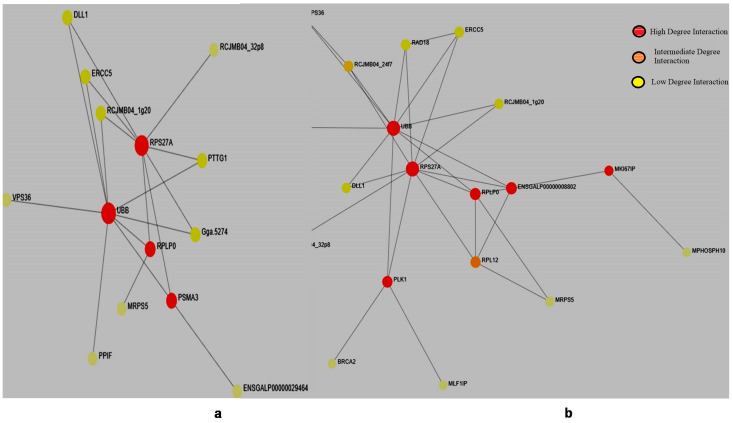
Protein–protein interaction network of genes with high impact variations. (**a**) Broiler. (**b**) *K. favorella*.

*Salmonella* infection affects every chicken breed; however, every breed has its own defense mechanism to counter the infection. In both the chicken breeds, we found similar hub genes; however, in the resistant breed (*K. favorella*) PLK1, MK1671P gene mutations were hyperactive, suggesting a possible role in this particular breed. Plk-1 is the member of the serine/threonine polio-like kinase family has a vital role in immune signaling [33]. Further PLK-1 interacts with BRCA-2 and MLF1P, which regulate autophagy, antigen presentation, immune response, angiogenesis and apoptosis [34,35] (Figure 5).

## 4. Discussion

*Salmonella*, one of the major infectious diseases in poultry, causes considerable economic losses in terms of mortality and morbidity, especially in countries that lack effective vaccination programs. Besides being resistant to diseases, indigenous chicken breeds are also a potential source of animal protein in developing countries. For understanding the disease resistance, an indigenous chicken line *K. favorella*, and commercial broilers were selected [1]. The severity of clinical symptoms, pathological manifestations and bacterial load post infection were used to assess the disease susceptibility and resistance.

An effective immune response against the invading pathogen requires a balance between the pathogen clearance and self-damage. However, this balance likely to change dynamically as the infection will progress. The host will destroy the invading pathogen at the initial stage of infection and towards the end, the host repairs the damage so that it can return to its original state. An effective host response is referred to as a balancing resistance and infection tolerance mechanism [36]. Indigenous chickens are genetically more diversified than commercial breeds due to their extensive history of breeding and improvement through methods remarkably distinct from those employed for commercial varieties. Therefore, it is essential to preserve regional chicken breeds as genetic resources in order to be prepared for unforeseen breeding demands in the future [37,38].

In our pilot study, we found the bacterial load in the resistant chicken breed (*K. favorella*) was lower than the bacterial load in the susceptible chicken breed (broiler). Our studies were in accordance with the previous studies, which showed the bacterial load in the local chicken was lower than other chicken breeds [39,40]. *K. favorella* showed minor lesions in the liver and spleen while the broiler chicken showed major necrotic lesions. This was in consensus with previous studies, which showed that the higher the bacterial count, the greater the pathological score [41].

The *K. favorella*, a well-known indigenous chicken breed from the north Indian state of Jammu and Kashmir, is regarded as the most important source of animal protein [13]. This native breed is disease-resistant and highly adapted to local climate circumstances, feed, and stress management [14]. For understanding the disease resistance mechanism, we have analyzed RNA sequencing data and performed a comparative study between *K. favorella* and broiler chicken breeds [1]. To evaluate putative polymorphisms in disease resistance genes, SNP detection analysis was performed using RNA seq data from 10 *K. favorella* and 10 broiler chicken.

A total of 1,141,122 and 1,151,874 variants were identified from *K. favorella* and broiler, respectively. A total of 1778 (1070 SNPs and 708 INDELs) and 1459 (859 SNPs and 600 INDELs) were identified in broiler and *K. favorella*, respectively. The KEGG and gene ontology analyses revealed that the genes were engaged in a variety of significant immune-related pathways. The MAPK signaling pathway, ECM-receptor interaction, Wnt signaling route, FoxO signaling pathway, and cellular senescence were shown to be substantially enriched in *Ka. favorella*. These pathways stimulate the immune response against *Salmonella* infection [42,43]. WNT signaling is essential for maintaining tissue homeostasis, epithelial barrier functioning, inflammatory cytokine production and modulation, host cell innate defense mechanisms, and the integration of innate and adaptive immunity [44]. In *K. favorella*, amplification of the WNT signaling pathway in response to *Salmonella* infection could increase B cell survival or proliferation [45]. Recent studies suggest that Wnt signaling performs an essential function in immune cell modulation and counteracts various disorders [46]. We found variations in different genes that regulate Wnt signaling (Figure S1) (TCF7, LRP5, CaMKII, WNT5A, NLK, etc.). TCF7 plays a vital role in tissue repair, remodeling and disease pathogenesis [47]. LRP5 has been found to possess a novel role in IL-10 signaling, thereby exerting a protective role during inflammation [48]. CaM-dependent proteins (CaMKII) have a critical role in infectious diseases through involvement in inflammatory processes, apoptosis and necroptosis [49]. Wnt5A promotes the death of numerous bacterial pathogens by altering actin assembly in macrophages, and thus resulting in bacterial phagocytosis [50]. Nemo-like kinase (NLK) has a role in modulating immune responses through regulation of NF-κB signaling by interfering with different signaling molecules [7]. In broilers, the enrichment analysis revealed that genes with high impact SNPs were involved mainly in metabolic pathways, fatty acid metabolism, carbon metabolism and amino acid metabolism (Arginine and proline metabolism). *Salmonella* utilizes these metabolites as an energy source for its intracellular survival and proliferation [51].

**Figure 6:**
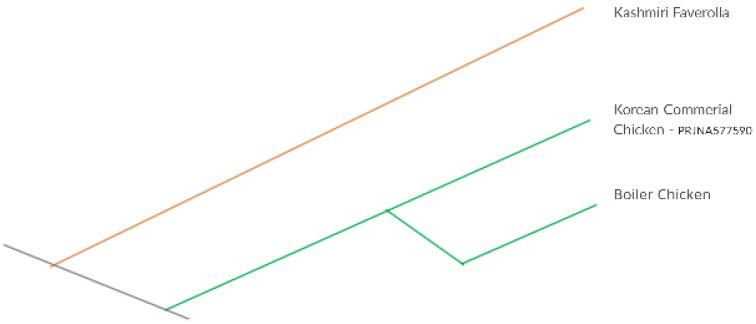
Comparative phylogenetic analysis between commercial and native chicken breeds.

The phylogenomic analysis revealed the exhaustive similarity between commercially available chicken breeds and possible similar mechanism of weak resistance against *Salmonella* infection (Figure 6). Resistance to salmonellosis in chicken greatly varies among the chicken line [52]. Due to breed differences, there is significant genetic heterogeneity in chicken for resistance to *S. typhimurium* [39].

MADPRT1, PPARD, IL18, IL18R1, TNFRSF10B, IL1R1, TNFAIP1, MMP28, SLC9A9, SLC5A10, SLC13A2 genes were identified with high impact SNPs involved in *Salmonella* disease resistance in chicken. This correlates with our previous study, which highlights their role in innate and adaptive immune responses [1]. Genetic variation in IL-18 has been associated with increased risk of atopy and asthma [53,54]. Further, IL-18 polymorphism has been linked to an increased or decreased progression of hepatocellular carcinoma [55]. Polymorphisms and haplotypes in TNFRSF10B are associated with an increased risk of death in non-small cell lung cancer [56].

## 5. Conclusions

SNP analysis demonstrated a significant difference between the *K. favorella* and broiler chickens in disease resistance. The high impact SNP variations in *K. favorella* and broilers were mostly engaged in metabolic pathways followed by the differential expression of some immune-related genes. Phylogenomic analysis based on the SNP studies showed clear genetic variations of the two breeds, based on the resistant mechanisms of indigenous poultry breeds (resistant) and susceptibility of broiler to *Salmonella* infection. These findings will offer fresh perspectives on the genetic diversity in chicken breeds and will aid in genomic selection of poultry birds.

## Figures and Tables

**Table 1:** List of genes with high impact SNPs involved in *Salmonella* disease resistance in chicken.

Gene	Chromosome	Reference Nucleotide	Mutated Nucleotide	SNP Nature	Impact
MADPRT1	1	C	T	Stop gained	HIGH
PPARD	26	A	T	Splice donor variant and intron variant	HIGH
IL18	24	G	A	Splice donor variant and intron variant	HIGH
IL18R1	1	TCC	TCCC	frameshift variant	HIGH
TNFRSF10B	22	A	C	Splice acceptor variant and intron variant	HIGH
IL1R1	1	T	A	Splice donor variant and Intron variant	HIGH
TNFAIP1	19	G	A	Stop gained	HIGH
MMP28	19	G	T	Splice donor variant and intron variant	HIGH
SLC9A9	9	T	A	Splice donor variant and intron variant	HIGH
SLC5A10	14	A	G	Stop lost and splice region variant	HIGH
SLC13A2	19	T	C	Splice donor variant and intron variant	HIGH

## Data Availability

The sequencing data used in the current study were downloaded from NCBI GEO database (Accession ID GSE168060).

## Supplementary Materials

The following supporting information can be downloaded at: https://www.mdpi.com/article/10.3390/genes14061283/s1, Figure S1: Genes with high impact (in Red) in Wnt signaling pathway; Table S1: Assessment of disease resistance and susceptibility by different parameters; Table S2: The chromosomal distribution of SNPs and INDELs; Table S3: High-impact SNPs and INDELs specific to *K. favorella*; Table S4: High-impact SNPs and INDELs specific to broiler chicken; Table S5: Pathways affected by high-impact SNPs in *K. favorella*; Table S6: Pathways affected by high-impact SNPs in broiler; Table S7: Gene Ontology analysis of genes with high impact SNPs and INDELs in *K. favorella*; Table S8: Gene Ontology analysis of genes with high impact SNPs and INDELs in broiler.

## Abbreviations

DNA	Deoxyribonucleic acid
mRNA	Messenger RNA
TCF	Transcription Factor
CaMK	CaM-dependent proteins
SSRs	Simple sequence repeats
INDELs	insertions and deletions
BRCA	Breast cancer gene
SNP	Single nucleotide polymorphism
KEGG	Kyoto encyclopaedia of genes and genomes
IL	Interleukin
NLK	Nemo-like kinase
LRP	Low-density lipoprotein receptor-related protein
GO	Gene ontology
MLF1P	Myeloid leukemia factor
