# Supplementary material for: Identification of SNPs Related to Salmonella Resistance in Chickens Using RNA-Seq and Integrated Bioinformatics Approach
Source: Genes (Basel). 2023 Jun 17;14(6):1283. doi: 10.3390/genes14061283 (PMC10297900; doi:10.3390/genes14061283)
Supplement: Supplementary file 1 [file genes-14-01283-s001.zip › Supplementary table.pdf]

Table S1: Clinical scoring of the infected *Kashmir favorella* and broiler chicken breeds (n=6). The data has been presented as Mean ±SD. The comparison of data was done by independent t test.

Parameter	Chicken Breed	Mean clinical score ±SD	F value	df	P-value
Appearance	Broiler	3.00±0.632	0.250	10	0.003
	Kashmir favorella	1.66±0.516		9.615	0.003
Behaviour with provocation	Broiler	3.00±0.894	0.160	10	0.111
	Kashmir favorella	2.1±0.752		9.61	0.111
Provoked behaviour	Broiler	3.66±0.516	0.625	10	0.004
	Kashmir favorella	2.50±0.547		9.71	0.004
Handling	Broiler	2.50±0.547	-	10	0.049
	Kashmir favorella	2.0±0.00		9.96	0.076
**Total Score**	**Broiler**	**12.16±0.147**	**2.25**	**10**	**0.00**
	Kashmir favorella	**8.33±0.816**		**7.81**	**0.001**

Table S2: Variant rate details

Chromosome	Length	Variants in broiler	Variants in *Kashmir favorella*
1	197,608,386	181,016	186,251
2	149,682,049	120,327	128,518
3	110,838,418	100,357	104,496
4	91,315,245	94,863	95,996
5	59,809,098	75,868	78,190
6	36,374,701	44,514	43,875
7	36,742,308	39,088	41,879
8	30,219,446	38,358	38,953
9	24,153,086	34,179	34,874
10	21,119,840	31,207	30,320
11	20,200,042	23,550	23,031
12	20,387,278	26,525	25,642
13	19,166,714	23,610	22,921
14	16,219,308	30,809	28,582
15	13,062,184	25,024	24,290
16	2,844,601	6,319	5,076
17	10,762,512	21,064	18,143
18	11,373,140	19,776	20,063
19	10,323,212	22,185	20,606

20	13,897,287	24,291	21,866
21	6,844,979	15,288	14,978
22	5,459,462	5,036	4,045
23	6,149,580	14,235	12,279
24	6,491,222	8,535	8,112
25	3,980,610	8,926	6,757
26	6,055,710	15,726	14,092
27	8,080,432	13,582	10,542
28	5,116,882	16,157	14,983
30	1,818,525	3,414	3,004
31	6,153,034	10,318	5,380
32	725,831	1,995	1,499
33	7,821,666	8,246	6,507

Table S3: SNP type in broiler chicken

**Type**	**Percent**
HIGH	0.091%
LOW	5.689%
MODERATE	1.968%
MODIFIER	92.252%

Type	Percent
MISSENSE	26.845%
NONSENSE	0.167%
SILENT	72.988%

Table S4: SNP type in *Kashmir favorella* chicken

Type	Percent
HIGH	0.076
LOW	5.467
MODERATE	1.812
MODIFIER	92.645

Type	Percent
MISSENSE	26.036
NONSENSE	0.178
SILENT	73.785

Table S5: Pathways affected by high-impact SNPs in *Kashmir favorella*

Pathway	ID	Genes	FE%	P-value	FDR[2]
MAPK signaling pathway	gga04010	17	6.51	0.00817	0.1352
ECM-receptor interaction	gga04512	8	9.63	0.00852	0.1393
Carbon metabolism	gga01200	9	8.82	0.00909	0.1406
Wnt signaling pathway	gga04310	11	7.80	0.00958	0.1457
FoxO signaling pathway	gga04068	10	8.13	0.01024	0.1505

Cellular senescence	gga04218	11	7.69	0.01050	0.1535
NOD-like receptor signaling pathway	gga04621	10	8.06	0.01076	0.1555
Phagosome	gga04145	12	9.09	0.00225	0.0676
Endocytosis	gga04144	17	7.45	0.00232	0.0676
Apoptosis	gga04210	11	8.66	0.00473	0.0972
Notch signaling pathway	gga04330	6	13.63	0.00494	0.1000
Herpes simplex virus 1 infection	gga05168	12	8.00	0.00583	0.1097
Protein processing in ER	gga04141	12	7.84	0.00673	0.1234
Ribosome	gga03010	15	12.5	2.74E-05	0.0047
Metabolic pathways	gga01100	70	5.42	5.41E-05	0.0071
Protein export	gga03060	6	26.08	0.00026	0.0177
Influenza A	gga05164	13	9.62	0.00092	0.0436
Cell cycle	gga04110	11	9.64	0.00220	0.0676
Tryptophan metabolism	gga00380	6	15.38	0.00289	0.0733
Lysine degradation	gga00310	7	12.96	0.00317	0.0767

Table S6: Pathways affected by high-impact SNPs in broiler.

**Pathway**	**ID**	**Genes**	**FE%**	**P-value**	**FDR[2]**
Metabolic pathways	gga01100	86	6.66	1.33E-06	0.0003
Herpes simplex virus 1 infection	gga05168	18	12.00	5.35E-05	0.0063
Fatty acid biosynthesis	gga00061	6	35.29	0.000148	0.0130

Influenza A	gga05164	16	11.85	0.000155	0.0132
Fatty acid metabolism	gga01212	9	16.98	0.000434	0.0288
Carbon metabolism	gga01200	12	11.76	0.001059	0.0548
Citrate cycle (TCA cycle)	gga00020	6	21.42	0.001394	0.0655
NOD-like receptor signaling pathway	gga04621	13	10.48	0.001742	0.0747
Arginine and proline metabolism	gga00330	7	16.66	0.002048	0.0775
Ribosome	gga03010	12	10.00	0.003669	0.0956
Adipocytokine signaling pathway	gga04920	8	12.50	0.005023	0.1140
Lysosome	gga04142	11	10.00	0.005289	0.1170
Cellular senescence	gga04218	13	9.09	0.005369	0.1170
Apoptosis	gga04210	12	9.44	0.00555	0.1175
Lysine degradation	gga00310	7	12.96	0.007107	0.1291
RNA transport	gga03013	12	9.09	0.007312	0.1311
Phagosome	gga04145	12	9.09	0.007312	0.1311
Protein processing in endoplasmic reticulum	gga04141	13	8.49	0.008906	0.1428
RIG-I-like receptor signaling pathway	gga04622	7	12.28	0.009205	0.1437
Endocytosis	gga04144	17	7.45	0.009984	0.1497

Table S7: Gene Ontology analysis of genes with high impact SNPs and INDELs in *Kashmir favorella*.

GO Term	GO ID	Genes	FE%	P Value
**Molecular Function**				
ATP binding	GO:0005524	86	8.01	2.51E-13
Histone binding	GO:0042393	17	17.34	1.42E-07
RNA binding	GO:0003723	32	8.76	1.73E-06
Protein C-terminus binding	GO:0008022	15	14.15	7.26E-06
Identical protein binding	GO:0042802	45	6.63	1.68E-05
DNA binding	GO:0003677	31	7.69	2.78E-05
Magnesium ion binding	GO:0000287	17	11.03	3.51E-05
Structural constituent of ribosome	GO:0003735	15	11.71	5.39E-05
Enzyme binding	GO:0019899	14	11.47	0.000116
Protein homodimerization activity	GO:0042803	31	7.07	0.000117
**Biological Processes**				
Negative regulation of cell population proliferation	GO:0008285	19	10.32	2.90E-05
Magnesium ion transport	GO:0015693	5	55.55	5.10E-05
Iron-sulfur cluster assembly	GO:0016226	6	35.29	6.50E-05
Nucleosome assembly	GO:0006334	7	25	1.00E-04
Ribosomal large subunit assembly	GO:0000027	6	31.57	0.0001
Translation	GO:0006412	13	12.14	0.0001
Neural tube closure	GO:0001843	9	16.98	0.0001

Response to virus	GO:0009615	7	23.33	0.0001
Negative regulation of transcription by RNA polymerase II	GO:0000122	34	6.53	0.0002
Negative regulation of phosphatidylinositol 3-kinase signaling	GO:0014067	4	57.14	0.0002
**Cellular Function**				
Nucleoplasm	GO:0005654	121	7.92	3.38E-18
Cytosol	GO:0005829	143	6.84	2.78E-16
Nucleus	GO:0005634	152	6.13	1.64E-13
Cytoplasm	GO:0005737	144	6.26	1.85E-13
Mitochondrion	GO:0005739	47	7.61	3.29E-07
Plasma membrane	GO:0005886	87	5.50	3.74E-06
Chromatin	GO:0000785	10	14.08	0.000248
Cul4-RING E3 ubiquitin ligase complex	GO:0080008	5	35.71	0.00026
Integral component of membrane	GO:0016021	117	4.56	0.00026
Protein-containing complex	GO:0032991	20	8.33	0.000273

Table S8: Gene Ontology analysis of genes with high impact SNPs and INDELs in broiler

GO Term	GO ID	Genes	FE%	P-Value
**Molecular Function**				
ATP binding	GO:0005524	101	9.41	1.98E-15
Magnesium ion binding	GO:0000287	21	13.63	2.20E-06
Unfolded protein binding	GO:0051082	13	19.69	6.12E-06

Chromatin binding	GO:0003682	30	10	6.18E-06
protein C-terminus binding	GO:0008022	16	15.09	1.13E-05
RNA binding	GO:0003723	33	9.04	1.50E-05
metal ion binding	GO:0046872	51	7.14	4.00E-05
DNA binding	GO:0003677	34	8.43	4.14E-05
Histone binding	GO:0042393	14	14.28	6.74E-05
mRNA binding	GO:0003729	17	11.48	0.00013
**Biological Processes**				
Negative regulation of cell population proliferation	GO:0008285	20	10.86	7.46E-05
T cell differentiation	GO:0030217	7	30.43	8.93E-05
Positive regulation of transcription, DNA-templated	GO:0045893	22	9.90	0.0001
Regulation of microtubule polymerization or depolymerisation	GO:0031110	5	45.45	0.0002
Fatty acid metabolic process	GO:0006631	7	25	0.0002
Protein folding	GO:0006457	11	14.86	0.0002
Protein phosphorylation	GO:0006468	21	9.29	0.0003
**Cellular Component**				
Cytosol	GO:0005829	174	8.32	3.64E-21
Nucleoplasm	GO:0005654	138	9.04	1.59E-19
Nucleus	GO:0005634	179	7.22	6.82E-16
Cytoplasm	GO:0005737	147	6.39	1.90E-09

Plasma membrane	GO:0005886	106	6.70	5.18E-08
Endoplasmic reticulum	GO:0005783	47	8.96	3.07E-07
Mitochondrion	GO:0005739	51	8.26	9.45E-07
Centrosome	GO:0005813	32	9.60	6.53E-06
