# Supplementary figures and images for: Identification of SNPs Related to Salmonella Resistance in Chickens Using RNA-Seq and Integrated Bioinformatics Approach
Source: Genes (Basel). 2023 Jun 17;14(6):1283. doi: 10.3390/genes14061283 (PMC10297900; doi:10.3390/genes14061283)

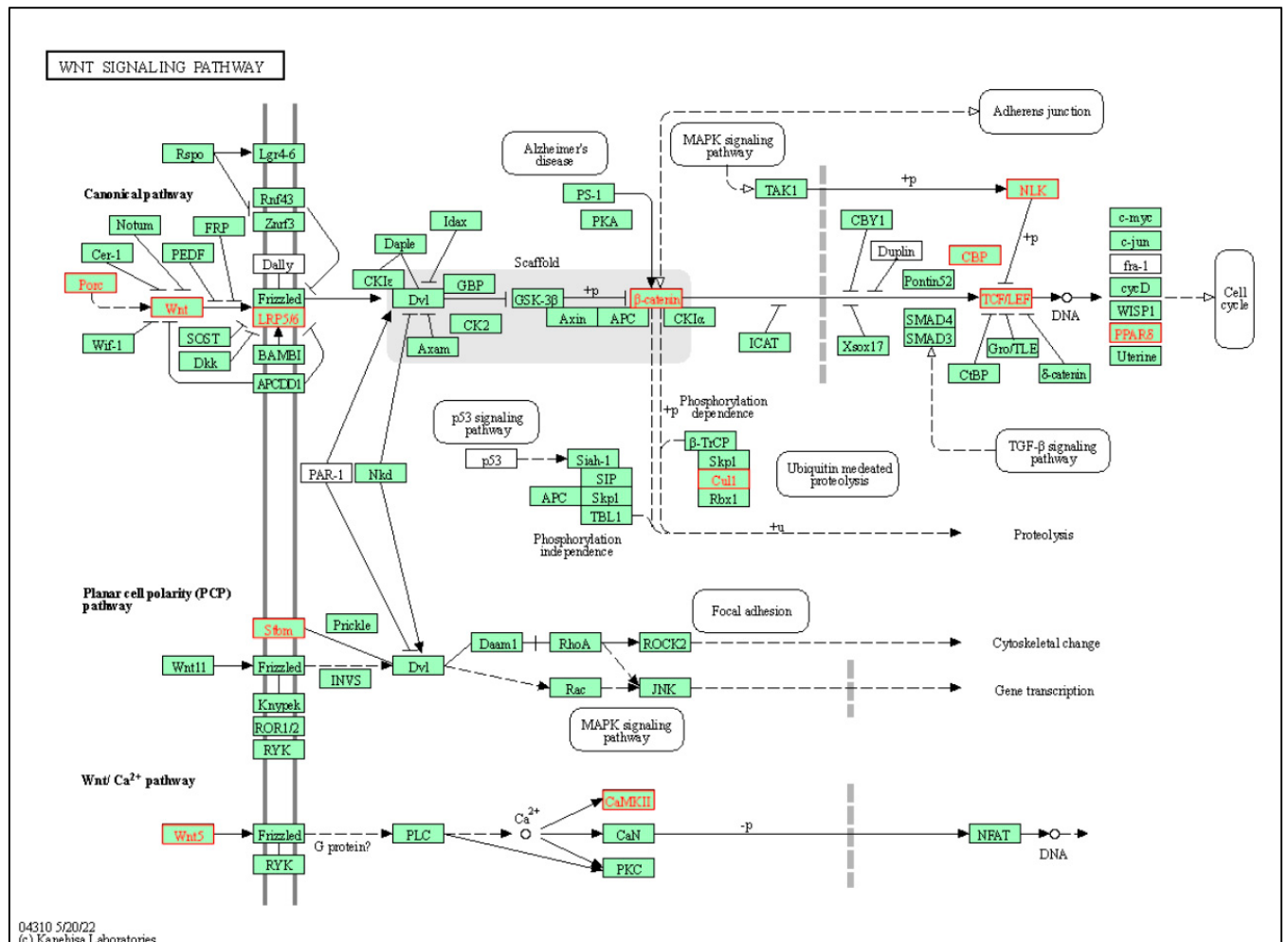

**Figure S1: Genes with high impact (in Red) in Wnt signalling pathway.**

Supplement: Supplementary file 1 [file genes-14-01283-s001.zip › Supplementary figure S1.pdf]
